# Supplementary material for: Dynalign II: common secondary structure prediction for RNA homologs with domain insertions
Source: Nucleic Acids Res. 2014 Nov 21;42(22):13939–48. doi: 10.1093/nar/gku1172 (PMC4267632; doi:10.1093/nar/gku1172)
Supplement: SUPPLEMENTARY DATA [file supp_gku1172_nar-02021-z-2014-File012.zip › manual/javadoc/RNAstructure_java_drawing/source/runners/package-summary.html]

RNAstructure\_java\_drawing.source.runners


JavaScript is disabled on your browser.


- Overview
- Package
- Class
- Tree
- Index
- Help

- Prev Package
- Next Package

- Frames
- No Frames

- All Classes

# Package RNAstructure\_java\_drawing.source.runners

A package that holds the run templates that are used when the Java drawing framework is wrapped into self-contained executables.

See: Description

- Class Summary

  | Class | Description |
  | --- | --- |
  |  |  |
  | --- | --- |
  | PlotRunner | A class that runs RNAstructure Java dot plot drawing independently of the main RNAstructure application. |
  | RunnerTemplate | A class that templates the way in which the RNAstructure Java drawing framework is run independently. |
  | StructureRunner | A class that runs RNAstructure Java structure drawing independently of the main RNAstructure application. |


## Package RNAstructure\_java\_drawing.source.runners Description

A package that holds the run templates that are used when the Java drawing framework is wrapped into self-contained executables.
  
The run templates are in a hierarchy, with the base class handling all common run tasks.

- Overview
- Package
- Class
- Tree
- Index
- Help

- Prev Package
- Next Package

- Frames
- No Frames

- All Classes
